# The Phospholipase A2 Superfamily: Structure, Isozymes, Catalysis, Physiologic and Pathologic Roles

**DOI:** 10.3390/ijms24021353

**Published:** 2023-01-10

**Authors:** Shibbir Ahmed Khan, Marc A. Ilies

**Affiliations:** Department of Pharmaceutical Sciences and Moulder Center for Drug Discovery Research, Temple University School of Pharmacy, 3307 N Broad Street, Philadelphia, PA 19130, USA

**Keywords:** phospholipase A2, structure, isozyme, kinetics, catalysis, activation mechanism, substrate specificity, subcellular localization, tissue distribution, physiology, pathology

## Abstract

The phospholipase A2 (PLA2) superfamily of phospholipase enzymes hydrolyzes the ester bond at the sn-2 position of the phospholipids, generating a free fatty acid and a lysophospholipid. The PLA2s are amphiphilic in nature and work only at the water/lipid interface, acting on phospholipid assemblies rather than on isolated single phospholipids. The superfamily of PLA2 comprises at least six big families of isoenzymes, based on their structure, location, substrate specificity and physiologic roles. We are reviewing the secreted PLA2 (sPLA2), cytosolic PLA2 (cPLA2), Ca^2+^-independent PLA2 (iPLA2), lipoprotein-associated PLA2 (LpPLA2), lysosomal PLA2 (LPLA2) and adipose-tissue-specific PLA2 (AdPLA2), focusing on the differences in their structure, mechanism of action, substrate specificity, interfacial kinetics and tissue distribution. The PLA2s play important roles both physiologically and pathologically, with their expression increasing significantly in diseases such as sepsis, inflammation, different cancers, glaucoma, obesity and Alzheimer’s disease, which are also detailed in this review.

## 1. Introduction

**Figure 1:**
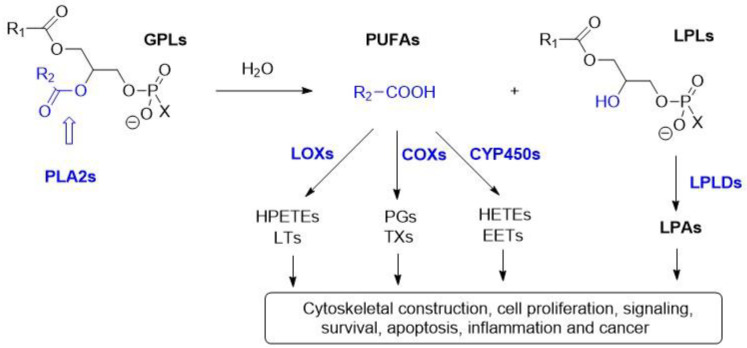
Schematic diagram of the fate of the phospholipids catalyzed by the PLA2s and further transformation into inflammatory mediators: PLA2s selectively hydrolyze the ester bond in sn-2 position of glycerophospholipids (GPLs) and release lysophospholipids (LPLs) and free fatty acids—mostly polyunsaturated fatty acids (PUFAs). PUFAs such as AA are metabolized by cyclooxygenases (COXs), lipoxygenases (LOXs) and cytochromes p450 (CYP450s) metabolic pathways into eicosanoids such as prostaglandins (PGs), thromboxanes (TXs), hydroperoxy-eicosatetraenoic acids (HPETEs), leukotrienes (LTs), hydroxy-eicosatetraenoic acids (HETEs) and epoxyeicosatrienoic acids (EETs). The LPLs can be further hydrolyzed by lysophospholipases D (LPLDs) into lysophosphatidic acids (LPAs) [3].

Phospholipase A2 (PLA2) belongs to the lipolytic family of enzymes that hydrolyze the ester bond at the sn-2 position of the phospholipids. Upon hydrolysis of the phospholipids, PLA2s release free fatty acids and generate lysophospholipids (LPLs). Free fatty acids such as arachidonic acid (AA) and oleic acid (OA) are important sources of energy [1]. Moreover, AA can be processed by cyclooxygenases (COXs), lipoxygenases (LOXs) and cytochrome p450 (CYP450) enzymes into eicosanoids, which can act as potent mediators of inflammation, cell signaling and carcinogenesis (Figure 1) [2,3]. On the other hand, lysophospholipids (LPL) contribute to phospholipid remodeling, membrane perturbation and cell signaling. Lysophospholipids are precursors of lysophosphatidic acid, being hydrolyzed by lysophospholipase D (LPLD), which plays an important role in cell proliferation, survival and migration (Figure 1) [4].

The phospholipase A2 enzymes are amphiphilic and quite different from the classical water-soluble enzymes, which act on water-soluble substrates [5]. The PLA2s act on phospholipid assemblies, which form supramolecular structures such as micelles, vesicles and liposomes in the aqueous environment. These PLA2s can only hydrolyze phospholipids when they are able to dock at the water/lipid interface of the vesicles via interfacial activation [6] (see below). 

The PLA2 superfamily has been classified into 16 groups (groups I to XVI), based on the chronology of their discovery [7]. The members of these PLA2 groups can also be divided into six subfamilies based on their location in the body, substrate specificity and differences in physiologic function (see Table 1), namely into secreted PLA2s (sPLA2s) (groups I, II, III, V, IX, X, XI, XII, XIII and XIV), cytosolic PLA2s (cPLA2s) (group IV), Ca^2+^-independent PLA2s (iPLA2s) (group VI), platelet-activating factor acetylhydrolase PLA2s (PAF-AH PLA2s) (groups VII and VIII), lysosomal PLA2s (LPLA2s) (group XV) and adipose-tissue-specific PLA2s (AdPLA2s) (group XVI) [6,7]. Among them, the first three subfamilies, namely sPLA2, cPLA2 and iPLA2, play critical roles in inflammation and cancer-related diseases. The PAF-AH PLA2, LPLA2 and AdPLA2 subfamilies have roles mainly in the development and progression of obesity and atherosclerosis [3,6,8].

## 2. Secreted PLA2 (sPLA2)

The secreted PLA2s were the first ones characterized and studied in detail as early as 1890 using the venom of cobras [7]. They were found as a major component of venoms from snakes, scorpions and other venomous reptiles. sPLA2s isolated from Old World snakes’ venom (cobras) were termed group I, and those from New World snakes’ venom (rattlesnakes) were classified as group II [17]. Both groups of enzymes contain seven disulfide bonds in their native fold. Among those, six disulfide bonds are common and are placed in similar locations, while the location of the seventh disulfide bond can vary between the two groups. Thus, group I PLA2s have the seventh disulfide bond formed between residues 11 and 77, while group II PLA2 has the disulfide bond between residues 50 and 138 [7]. The most noticeable difference between groups I and II is represented by a group of 6–7 additional amino acid residues at the C terminal of group II, which are absent in group I. 

**Figure 2:**
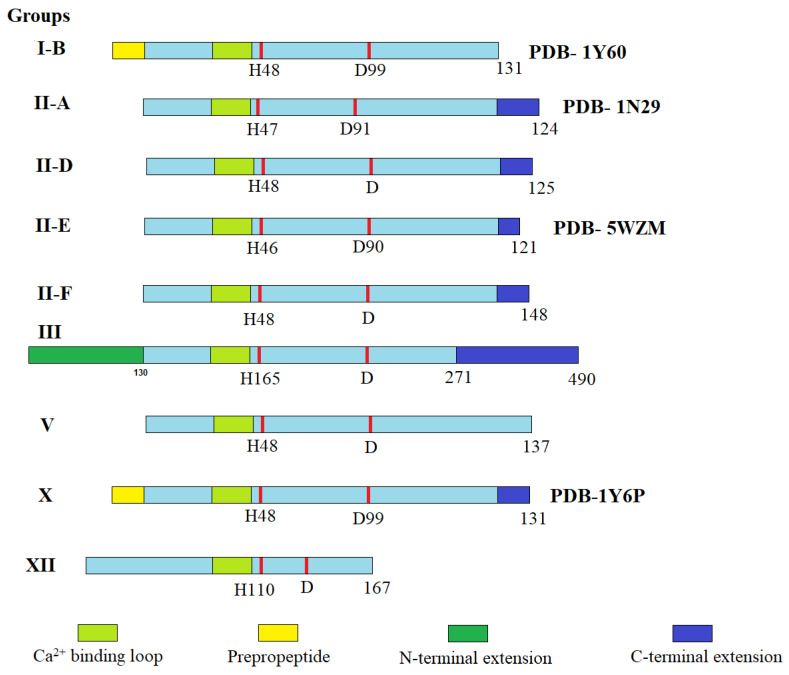
Schematic presentation of the different groups of secreted PLA2s found in humans (adapted from [6]). The Ca^2+^-binding loop is shown in light green, the key active site residues (His and Asp) are shown as red bars, and N-terminal (deep green) and C- terminal (deep blue) extension residues are also highlighted. Adapted with permission from [6]. Copyright 2011, American Chemical Society.

These C terminal amino acids are negatively charged, which makes the representatives of group II enzymes more negatively charged than group I enzymes [18]. The differences among different groups of sPLA2’s primary structure present in the human body are shown schematically in Figure 2. sPLA2s that were discovered in mammalian tissues or secreted pancreatic juices were similar to that of the Old World snakes’ PLA2 and were classified as group I, specifically group IB. The sPLA2s discovered in the human synovial fluid had more structural similarities to the New World snakes’ sPLA2s and were included in group II enzymes (Table 1) [17].

**Figure 3:**
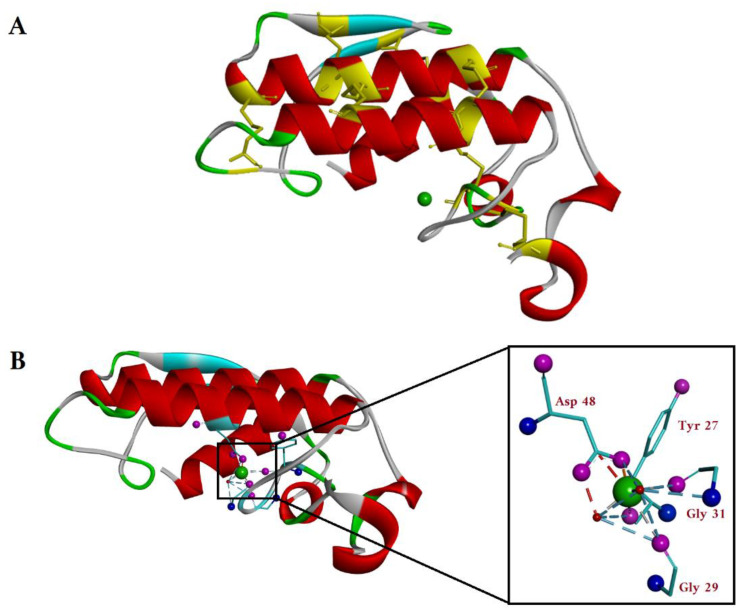
(**A**) Top view of the crystal structure of G-IA sPLA2 (*Naja naja*) (PDB-1PSH). The α-helices and β-sheets are shown in red and light blue. The cysteines forming the seven disulfide bonds are shown in yellow, also marking in yellow their position on the protein backbone. The calcium ion is shown as a green sphere in the enzyme active site. (**B**) Side view of the crystal structure of the sPLA2 with the Ca^2+^ coordination shown in detail in the zoomed insert. The oxygen atoms and the nitrogen atoms of the amino acids coordinated with Ca^2+^ (green sphere) are colored pink and blue, respectively. The Ca^2+^ is directly coordinated with carboxy group from Asp48 of one α-helix and by the C=O backbone groups of Tyr27, Gly29 and Gly31 from the opposite loop. (Image generated using BIOVIA Discovery studio.)

In mammals, the sPLA2 family contains ten catalytically active isoforms namely IB, IIA, IIC, IID, IIE, IIF, III, V, X and XII [6]. As mentioned above (Table 1), sPLA2s are low-molecular-weight enzymes, with a molecular weight ranging from 13 to 19 kDa and containing seven disulfide bonds (yellow). All the isoforms of sPLA2 have three long α-helices (red), two-stranded β-sheets (light blue) and a highly conserved Ca^+2^-binding loop (green sphere) as common structural features (Figure 3A). 

Their active site has a His/Asp catalytic dyad [6]. Thus, the enzyme polarizes a Ca^2+^-bound H_2_O molecule with the help of His and Asp residues from the catalytic site, which will subsequently attack the carbonyl group at the sn-2 position of phospholipids. The Ca^+2^ ion stabilizes the transition state by coordinating the carbonyl group and the negative charge from the phosphate group [6]. Therefore, Ca^2+^ is needed for sPLA2 in the catalysis act. 

### 2.1. sPLA2 Catalytic Mechanism

A Ca^2+^ ion is required for both binding of the substrate and for catalysis. This Ca^2+^ ion is hepta-coordinated with a pentagonal bipyramidal coordination geometry (Figure 3B) [19]. Among the seven coordinated bonds, five of the ligands are provided by the protein backbone: one bidentate Asp48 side chain carboxylic group from an α-helix loop and three C=O backbone groups of Tyr27, Gly29 and Gly31 from the calcium-binding loop. The other coordinating ligands are two water molecules, one in the axial region and the other one in the equatorial region (Figure 3B) [19].

**Figure 4:**
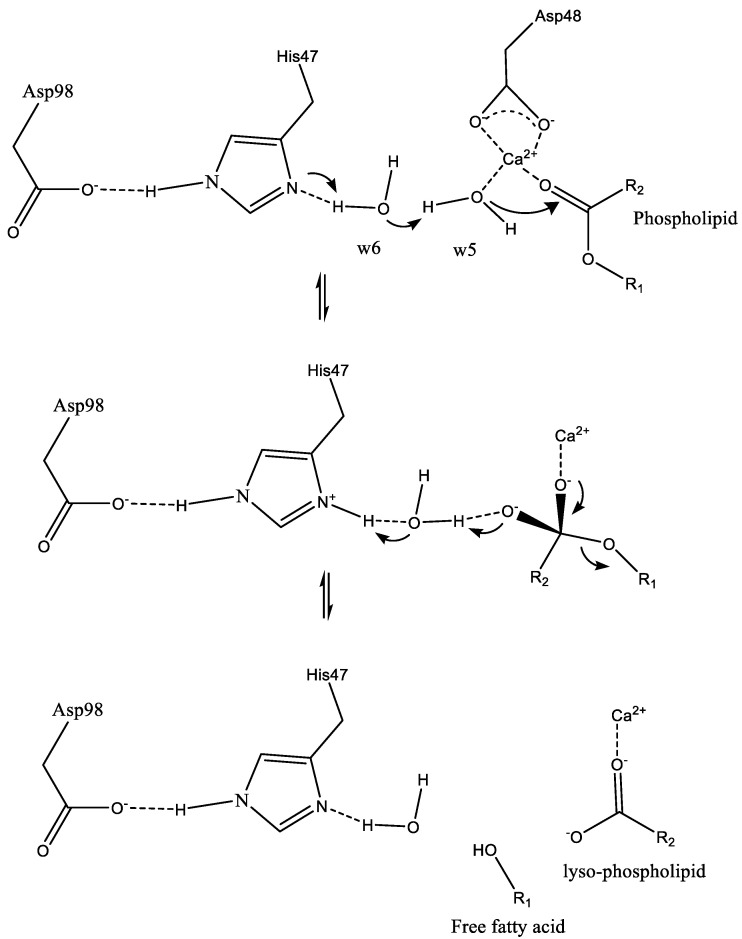
Proposed catalytic mechanism for sPLA2. Asp98 and His47 constitute the catalytic dyad which plays a key role in catalysis. These two residues are hydrogen-bonded together and further connected with the Ca^2+^ ion with the help of two water molecules (w5 and w6). His47 facilitates the deprotonation of w5 water molecule, subsequently attacks the sn-2 carbonyl group of the substrate and forms the tetrahedral intermediate. The rate-limiting step is the decomposition of the tetrahedral intermediate [20].

The water molecule coordinated to the Ca^2+^ ion in the axial region is replaced by the sn-3 phosphate group of the substrate (not shown in Figure 4). Upon binding to the enzyme, the equatorial water molecule coordinated to the Ca^2+^ ion (w5) is polarized and deprotonated by His47, generating a hydroxyl ion, which then attacks the sn-2 ester carbonyl group and forms a tetrahedral intermediate (Figure 4). 

His47 residue becomes protonated via the neighboring water molecule (w6), which makes a proton wire between the His residue and the equatorial water (w5). This deprotonation via a water molecule bridge lowers the activation energy for the tetrahedral intermediate formation step. The decomposition of the tetrahedral intermediate is the rate-limiting step of the enzymatic reaction [20].

### 2.2. Interaction of sPLA2 with Phospholipid Substrate

**Figure 5:**
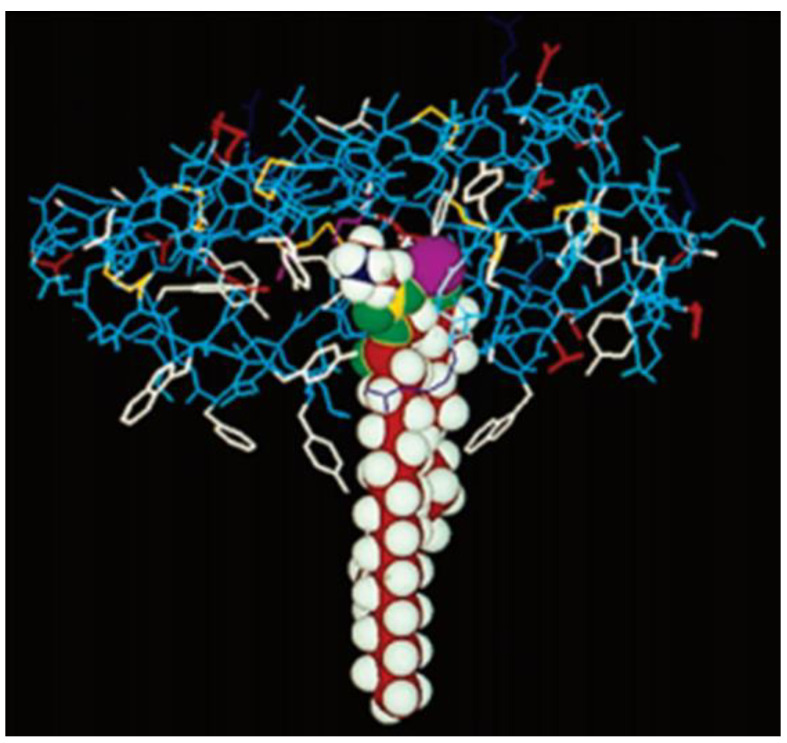
Cartoon depicting the X-ray crystal structure of cobra venom sPLA2 (wireframe) with dimyristoyl phosphatidylethanolamine (shown in a space-filling model) bound to it. The active site dyad His48/Asp99 and the Ca^2+^ ion are depicted together as a purple sphere. The aromatic interfacial residues Phe-5, Trp-19, Tyr-52 and Tyr-69 of PLA2 are shown in white. These residues of the enzyme are reaching as deep as 9–10 carbons in the acyl chain of the fatty acid while the rest of the chain is submerged into the lipid bilayer. Reprinted with permission from [21]. Copyright 1994, Elsevier.

It is very difficult to obtain crystals of sPLA2 in the presence of phospholipids. To elucidate the bindings of the protein to the self-assembled phospholipids in a bilayer, the X-ray crystal structure of the sPLA2 from the native cobra venom was modeled in a cartoon representation of phospholipid substrate, using a space-filling model, to reveal how the sPLA2 enzyme might interact with the substrate. Only 9–10 carbon atoms of the sn-2 acyl chain of the lipid interact with the enzyme, while the rest of the chain remains buried in the lipid bilayer (Figure 5) [21].

### 2.3. Interaction of sPLA2 with Phospholipid Bilayers: Coarse-Grained Molecular Dynamic (CG-MD) and Atomistic Molecular Dynamic (AT-MD) Simulation Results

**Figure 6:**
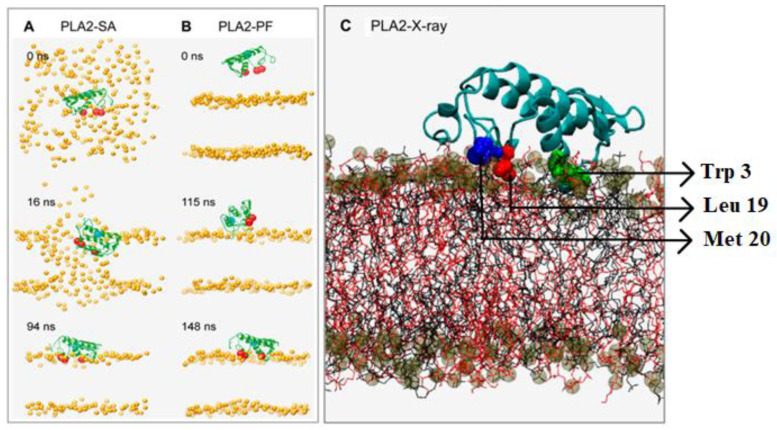
Snapshots from the CG-MD simulations at different timeframes: (**A**) sPLA2-SA (self-assembled simulation); (**B**) sPLA2-PF (preformed simulation). The backbone of the enzyme is shown in green; the hydrophobic anchoring residues are shown in red; and the active site residue (H48) is depicted as a blue sphere; (**C**) snapshot of atomistic MD simulation of sPLA2 (PLA2-X-ray) on phospholipid bilayer. The protein backbone is depicted in light blue, and the three hydrophobic residues Trp-3, Leu-19 and Met-20 are colored in green, red, and deep blue, respectively, in a space-filling model. Reprinted/adapted with permission from [22]. Copyright 2008, Elsevier.

The coarse-grained molecular dynamic (CG-MD) simulations can generate long simulation times and therefore enable one to optimally locate a surface-active enzyme. The technique was applied to study the interaction of secreted phospholipase A2 (sourced from the porcine pancreas) with the lipid bilayers made out of POPC and POPG [22]. The simulation results are shown in Figure 6, with the sPLA2 enzyme backbone shown in green, the hydrophobic residues depicted as red spheres and the active site residue (His-48) presented as blue spheres. The phospholipid polar heads are shown as orange spheres [22]. 

Two different simulation protocols were run with a duration of 200 ns each (Figure 6). The interaction of sPLA2 with the lipid self-assembly was the focus of the first simulation (PLA2-SA), while in the second simulation the bilayer was self-assembled in the presence of PLA2 (Figure 6A). The lipid and the water molecules were randomly assigned surrounding PLA2. Alternatively, the preformed bilayer simulations (PLA2-PF) were performed, where the PLA2 molecule was initially placed with its center of mass approximately ~30 Å from the surface of the bilayer (Figure 6B). 

Both simulations produced similar results, with the sPLA2 enzyme positioning itself in a similar orientation at the water/lipid interface. The CG-MD simulations demonstrated that the sPLA2 was able to diffuse laterally with a diffusion coefficient of 6 (±3) × 10^−7^ cm^2^/s into the planar bilayer membrane, and the lipid substrates repacked themselves surrounding the enzyme, once it progresses [22]. 

Another useful computational tool is the atomistic molecular dynamics (AT-MD) simulation, which is used to study the conformational dynamics of integral membrane proteins. This simulation was used in the case of sPLA2 to study the protein’s ability to penetrate the lipid bilayer, with the results presented in Figure 6C [22]. The protein is shown in light blue color with the three hydrophobic anchor residues Trp-3, Leu-19 and Met-20 shown in green, red and blue. The POPC and POPG lipid chains are shown in black and red, and their phosphate groups as shown as brown and gray spheres, respectively. This AT-MD simulation showed that ~15–20 hydrogen bonds were made between sPLA2 and the lipid bilayer, and ~5–8 hydrogen bonds were made between the basic residues (Arg-6, Lys-10, Lys-122) and the carbonyl oxygens of lipid substrates [22]. 

The distances between the center of mass of the hydrophobic anchor residues Trp3, Leu9, Met20 (believed to stabilize the enzyme in the bilayer environment) and the center of mass of the lipid bilayer, as well as between the center of the mass of His-48 (catalytic residue) and the center of mass of the lipid bilayer, were measured to assess the depth of penetration of sPLA2 into the lipid bilayer. The results of the CG-MD simulation (Figure 6C) revealed that the hydrophobic anchors were at an average distance of ~20–4 Å from the bilayer center and that the catalytic site residue His-48 was located at an average distance of ~30 Å from the bilayer center, suggesting that the His residue was just above the bilayer surface (Figure 6C) [22]. 

**Figure 7:**
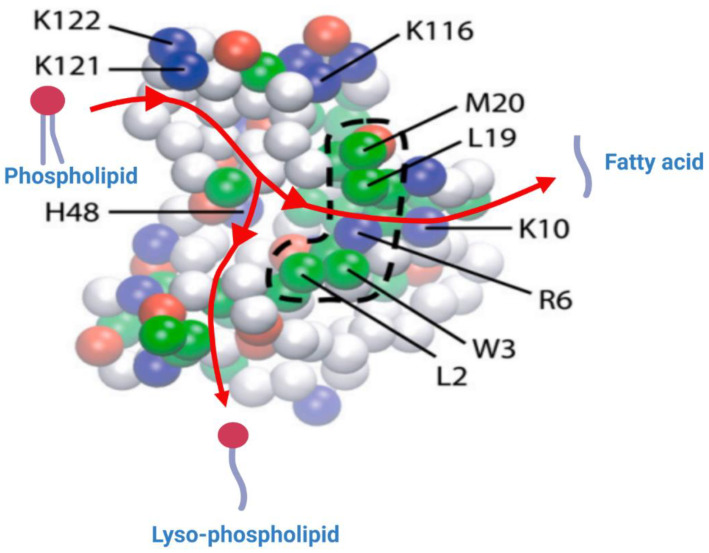
Representation of the bottom (inter)face of the sPLA2, which remains in contact with the lipid bilayer, generated through the CG simulation. The amino acid residues which remained deep in the bilayer are represented in strong colors, and the ones that are away from the bilayer are shown in faded colors. The hydrophobic, basic, acidic and polar residues are shown as green, blue, red and white spheres, respectively. The broken line delimiting the cluster of hydrophobic residues (Trp-3, Leu-19, Met-20) highlights the key residues believed to anchor the sPLA2 into the lipid bilayer. The basic residues (Arg-6, Lys-10, Lys-116, Lys-121 and Lys-122) form electrostatic interactions with the phosphate group and with the carbonyl group of the lipid substrate. Reprinted/adapted with permission from [22]. Copyright 2008, Elsevier.

The surface of the protein that binds to the membrane was found to be the same in both simulations. This surface has a collection of hydrophobic residues (Leu-2, Trp-3, Leu-19 and Met-20) surrounded by basic residues (Arg-6, Lys-10, Lys-116, Lys-121 and Lys-122) and polar residues (Figure 7) [22]. The residues Arg-6, Lys-10 and Lys-116 are believed to hydrogen bond with the lipid carbonyl oxygens and residues Lys-121, and Lys-122 are believed to form an electrostatic interaction with lipid phosphate moieties. It is quite obvious that the enzyme has a perfectly adapted interface and can dive significantly into the lipid bilayer. A general pathway for a phospholipid substrate’s pathway into the hydrophobic core of the enzyme can be hypothesized judging by the distribution of amino acid residues inserted into the lipid bilayer. A cartoon representation of this pathway is shown in Figure 7 (adapted and modified from [22]). The lipid substrate is believed to enter the enzyme and anchor its phosphate group electrostatically with Lys-121 and Lys-122, with the ester group located near the active site His-48. After hydrolysis, the fatty acid product comes out of the active site following a path defined by Arg-6 and Lys-10, and it is released into the bilayer. The lyso-PL leaves the active site through a different pathway, with the help of hydrophobic moieties (Leu-2, Trp-3), separately from the fatty acid (Figure 7).

### 2.4. sPLA2 Substrate Binding and Interfacial Kinetics

**Figure 8:**
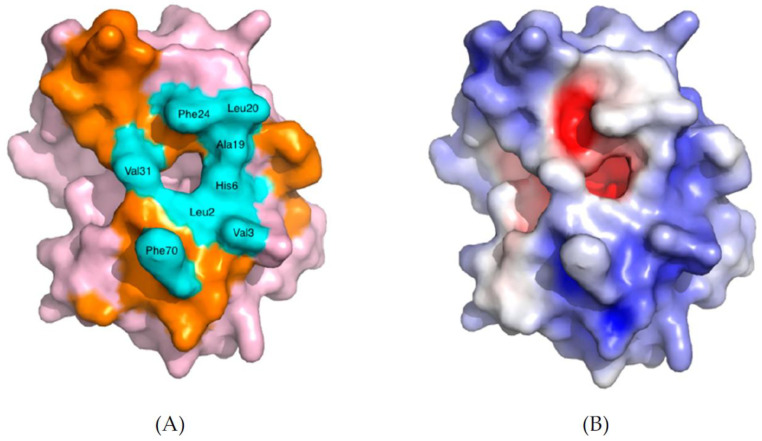
Space-filling model of human group IIA sPLA2 (PDB—3U8B). (**A**) The amino acids which form the i-face are colored orange, and the ones which create the entrance are highlighted in cyan blue, while the rest of the protein is shown in pink. (**B**) The electrostatic charge distribution is depicted either in blue (positive charge) or in red (negative charge). The white areas charge neutrality (non-polar), which corresponds to the hydrophobic entrance of the left figure. Reprinted from [26].

sPLA2s are interfacial enzymes and act at the lipid/water interface of the phospholipid self-assembled structures in water (micelles, liposomes, etc.). All the sPLA2s have a common hydrophobic pocket about 15 Å deep into the active site, where a single phospholipid substrate can position itself next to the catalytic residues for achieving the hydrolysis of the ester bond [23]. It is believed that the enzyme displaces water molecules from the region where it docks and creates an amphiphilic microenvironment. This special environment allows for the diffusion of the phospholipid substrate into the active site. The area surrounding the opening of the active site pocket of the protein forms the interfacial surface of the enzyme, known as the i-face. This i-face remains in direct contact with the membrane phospholipids and has a planar surface composed of approximately 20 amino acids and a total surface area of about 1500 Å (Figure 8) [24,25]. These amino acids in the i-face surface are mostly hydrophobic and amphiphilic and create strong interactions with the lipid bilayer needed for interfacial binding. Notably, some of the amino acids are cationic and can interact electronically and through hydrogen bonds with anionic substrates. As stated before and represented schematically in Figure 7, the amino acids within the i-face form a pathway into the active site (Figure 8A), through which a single phospholipid molecule can enter the active site and be hydrolyzed. The electron density map shows that the outer area of the enzyme is more basic (blue) in Figure 8B, while the inner core is acidic (red) in Figure 8B [26]. 

**Figure 9:**
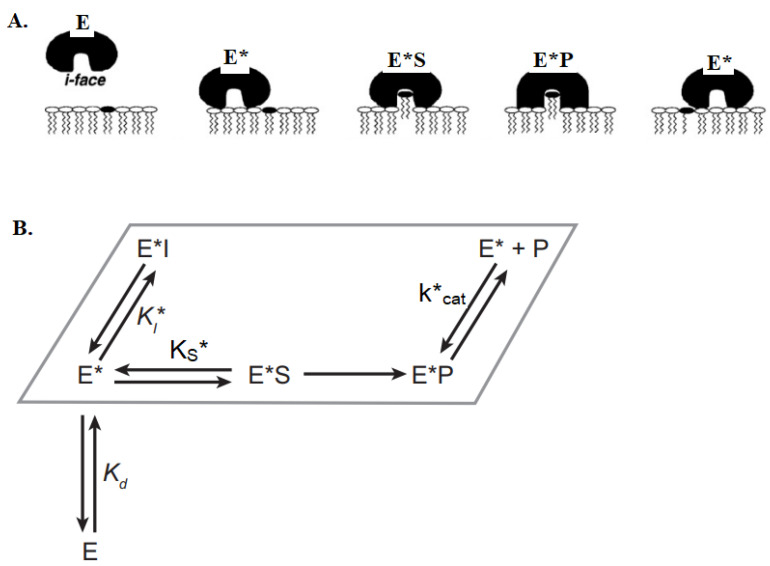
Schematic representation of interfacial binding and catalytic action of sPLA2. (**A**) The binding of the sPLA2 at the lipid/water interface through the i-face is essential for catalysis. The enzyme passes along the horizontal plane on the bilayer, while hydrolyzing the phospholipid molecules and releasing the LPL and FA products [20]. (**B**) The minimal kinetic scheme for the catalytic cycle of sPLA2 is shown in a parallelogram box that represents the lipid bilayer. Here, E denotes enzyme in the aqueous phase, E* denotes enzyme in a membrane-bound state without substrate and K_d_ is the interfacial dissociation constant for the enzyme at the interface. K_S_, K_cat_ and K_I_ are the dissociation constants for substrate, product and inhibitor, respectively. E*S and E*P denote the enzyme-bound substrate and enzyme-bound product, respectively. Adapted with permission from [20]. Copyright 2001, American Chemical Society.

When the enzyme binds to the bilayer’s interface with the help of its i-face surface, it undergoes an allosteric structural modification denoted as E* (Figure 9A). Subsequently, the enzyme pulls one phospholipid molecule, hydrolyzes it, releases the products and then moves to the next lipid substrate. Substrate specificity for sPLA2 depends on two factors: first is the ability of the enzyme to bind to the membrane surface (E → E*, characterized by an equilibrium dissociation constant, K_d_) (Figure 9B) and second is the rate of hydrolysis of different substrates by E* once bound at the membrane interface. This is controlled by the relative interfacial specificity constant (k_cat_*/K_S_*). Multiple catalytic cycles can occur without the desorption of the enzyme back into the aqueous phase since it binds to the interface tightly (scooting mode) (see below [20]). The interfacial binding is crucial for catalysis, though the binding energy can vary based on the nature of the vesicles. For example, the dissociation constant (K_d_) of porcine pancreatic PLA2 on anionic vesicles is <0.1 pM, whereas on zwitterionic vesicles it is more than 10 mM [27]. This might explain why some sPLA2s show substrate specificity on anionic vesicles over the zwitterionic vesicles.

### 2.5. Scooting vs. Hopping Mode of Action of sPLA2

**Figure 10:**
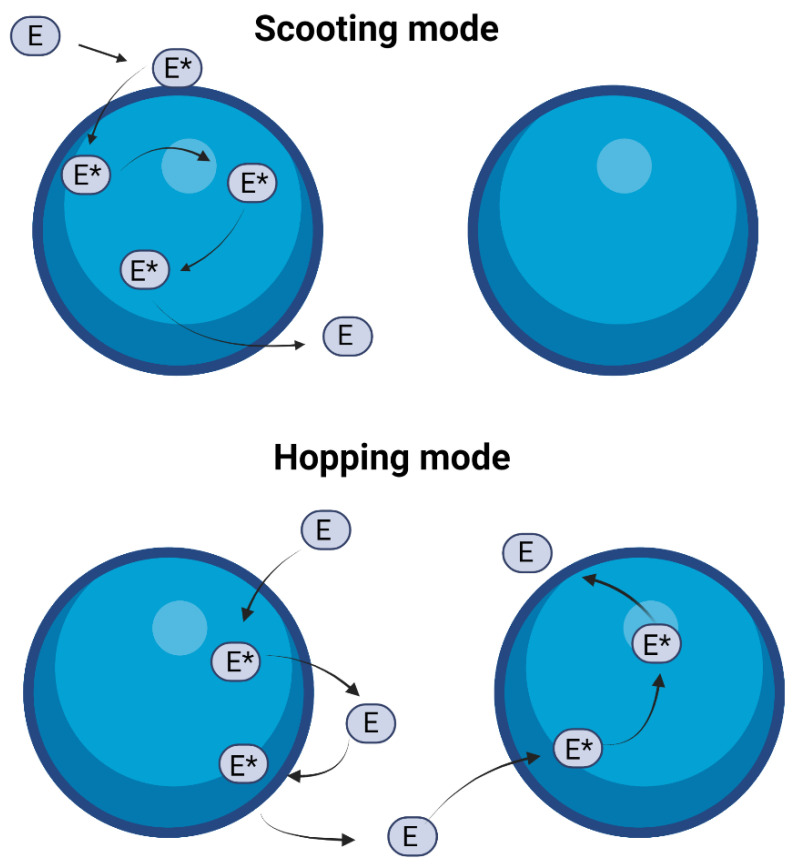
Cartoon showing the two main modes in which sPLA2 enzyme may interact with the lipid vesicles. In scooting mode (**top**), the enzyme does not exchange vesicles after each catalytic turnover, and in hopping mode (**bottom**), the enzyme leaves the interface (of the vesicle) after each catalytic cycle and moves to another vesicle. Adapted with permission from [20]. Copyright 2001, American Chemical Society. Created with BioRender.com.

The interaction of sPLA2 with the lipid vesicles can be explained considering two different modes of action (Figure 10). First, in the scooting mode, the enzyme does not leave the vesicles after each catalytic turnover but continues its catalytic cycle on the already docked vesicle. All the enzyme-containing vesicles behave similarly if the number of enzymes per vesicle remains the same. Alternatively, in the hopping mode, the enzyme dissociates from one vesicle after each catalytic turnover and moves to another residue. All the vesicles will hydrolyze uniformly as an interaction between the enzyme and the vesicle occurs homogeneously. It is hard to evaluate in which particular mode the enzyme might work on a given vesicle. The residence time of the enzyme at the interface is not constant over the time course as the reaction progresses [20]. 

## 3. Cytosolic PLA2 (cPLA2)

The cytosolic PLA2s (cPLA2s) are intercellular enzymes that belong to group IV of PLA2 family enzymes. This group of enzymes consists of six subgroups (A, B, C, D, E and F), which are also denoted as cPLA2α, cPLA2β, cPLA2γ, cPLA2δ, cPLA2ε and cPLA2ζ, respectively. The molecular weights of these cPLA2s range from 85 to 114 kDa [28]. These enzymes are mostly found in platelets, macrophages and tissues of liver, pancreas, brain and heart [6]. The first subgroup cPLA2α was discovered in human platelets and neutrophils in 1986 [29,30]. It is also the most studied cPLA2 enzyme. Most of the phospholipids such as phosphatidylcholine, phosphatidylethanolamine and phosphatidylinositol are substrates of cPLA2s. Moreover, this enzyme has high specificity for phospholipids containing arachidonic acid (AA) at the sn-2 position [6]. 

### 3.1. cPLA2 Crystal Structure

**Figure 11:**
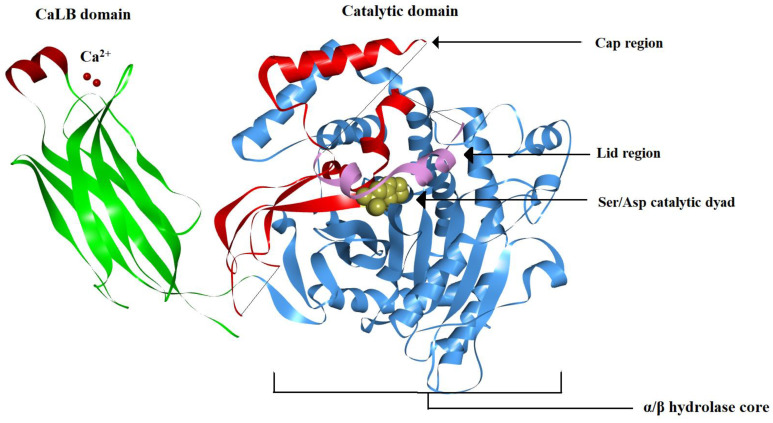
Ribbon diagram of a typical cPLA2 (PDB: 1CJY). The Ca^2+^-dependent lipid-binding (CaLB) domain is shown in green on the left side of the figure, with the two bound Ca^2+^ ions (required for protein translocation to the membrane) shown as red spheres. The catalytic domain is shown on the right side, the α/β hydrolase core is shown in blue and the catalytic dyad Ser/Asp is shown in yellow. The enzyme cap is shown in red, and the lid region covering the active site funnel is colored pink. The lid region prevents the lipid substrates to come in contact with the active site [31]. (Image generated using BIOVIA Discovery studio.)

The enzyme consists of two functionally distinct domains attached together via a flexible hinge: a Ca^2+^-dependent lipid-binding domain (CaLB), also known as the C2 domain, and a catalytic domain (Figure 11) [31]. This CaLB domain contains eight anti-parallel β-sheets folded into a sandwich-like structure. The catalytic domain has an active site consisting of a Ser/Asp dyad. The catalytic domain consists of 14 β strands and 13 α helices, respectively. Among them, 10 β-sheets and 9 α-helices form the central core of the catalytic domain (α/β hydrolase core). The remaining four β strands and the four α-helices (residues 370–548) divert from the central core, forming a ‘cap’ region. Within the ‘cap’ region, there is a string of amino acids (residues 413–457), which blocks the lipid substrate from entering the active site of the inactive form of the enzyme, acting as a closed ‘lid’. The lid is organized into a small loop, followed by a small helix (Fc) and a short turn. The X-ray crystal structure of the cPLA2 showed that the residues from 408–412 and the residues 434–457 do not have traceable electron density, suggesting that these flexible regions form the ‘lid hinges’ which can change its conformation when it comes in contact with the lipid substrate [31,32]. Both the CaLB domain and the catalytic domain are required for the full activity of the enzyme. Thus, for cPLA2 to be activated, it needs to translocate to the interfacial surface of the membrane. This is achieved through the recruitment of two Ca^2+^ ions, which bind to the CaLB domain and facilitate the anchoring of the enzyme to the lipid bilayer [31,33]. Unlike the sPLA2s, here the Ca^2+^ ion does not contribute to the catalytic activity in the cPLA2 family of enzymes. The catalytic domain does not penetrate deeply into the lipid bilayer, but it remains in close contact with the membrane surface through specific electrostatic interaction with the membrane phospholipids [31]. The cap region is rich in positively charged amino acid residues, which are hypothesized to make electrostatic contacts with the negatively charged groups of membrane phospholipids. When the enzyme is inactive, the lid covers the active site funnel and prevents any lipid substrate from reaching the active site. Upon binding to the lipid bilayer, the enzyme is activated and the lid opens up, allowing the binding of lipid substrates with the active site and the activation of the enzyme [32]. The active site funnel has an opening of approximately 7 Å in diameter with a depth of around 8 Å from the opening of the funnel down to the active site Ser228. The funnel is lined with hydrophobic residues which can hold the fatty acyl chain of the membrane phospholipids. The more prominent theory is that this funnel can relocate the phospholipid substrate from the membrane and lift it until the first cis double bond of the arachidonyl-bound phospholipid substrate reaches the surface of the membrane. This ‘lifting’ process also explains the cPLA2s preference for arachidonic-acid-containing phospholipids [31]. 

### 3.2. cPLA2 Activation Mechanism

**Figure 12:**
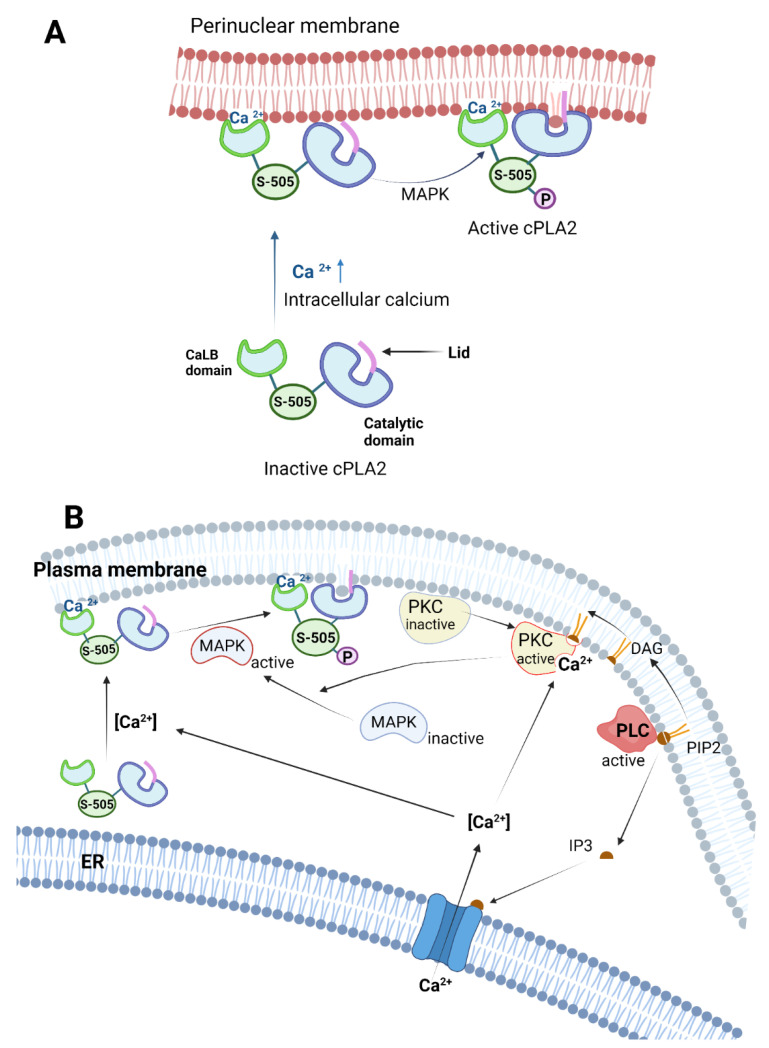
Multiple activation pathways for cPLA2 via calcium, phosphorylation and secondary messengers. (**A**) Ca^2+^ ion helps in binding the CaLB domain of the cPLA2 with the membrane; however, the catalytic domain is not oriented in the right direction for hydrolyzing the phospholipid substrate. Phosphorylation in the flexible linker of the cPLA2 via MAPK induces optimal conformation of the catalytic domain, which facilitates partial penetration of the catalytic domain into the membrane. (**B**) The schematics for multiple regulation pathways for cPLA2. PIP2 is hydrolyzed by PLC to form secondary messengers such as IP3 and DAG. IP3 opens up the Ca^2+^ ion channel in the perinuclear membrane of the ER, which increases the intracellular Ca^2+^ concentration. The DAG and the Ca^2+^ simultaneously activates PKC, which subsequently activates MAPK. The activated MAPK phosphorylates the cPLA2, which further enhances the cPLA2 activity. Created with BioRender.com.

cPLA2s are activated via several mechanisms, predominantly via Ca^2+^ ion binding, phosphorylation of specific cPLA2 residues and interaction with secondary messengers such as ceramide-1 phosphate (C1P) and phosphatidyl inositol bisphosphate (PIP2) (Figure 12) [6]. Thus, when the intracellular Ca^2+^ ion concentration [Ca^2+^] increases, two Ca^2+^ ions will bind to the CaLB domain and, as a result, the cPLA2 will translocate from the cytosol to the membrane region (plasma, perinuclear, etc.) [31]. The calcium-binding domain of the cPLA2 forms an anionic hole, which is neutralized by the two bound Ca^2+^ ions. This calcium-binding rigidifies the CaLB domain and optimizes the local charge and backbone conformation of the cPLA2, facilitating the interaction with the phospholipid membrane [34]. Additionally, phosphorylation of cPLA2 in the residues located in the flexible linker between the CaLB domain and catalytic domain increases both the binding affinity and the catalytic efficiency of the enzyme. This phosphorylation causes a conformational change of the interdomain region of the enzyme, moving the catalytic domain closer to the membrane surface (Figure 12A) [35]. A recent study showed that phosphorylation of cPLA2 at the Ser505 residue located in the flexible linker is another important factor for translocation of the cPLA2 to the bilayer membrane. Mutation of the Ser505 residue showed delayed response in translocating the enzyme to the membrane even at elevated levels of Ca^2+^ and PIP2, suggesting that phosphorylation of Ser505 works in tandem and probably helps Ca^2+^-mediated activation [36]. Multiple kinase enzymes are responsible for the phosphorylation of cPLA2, but it is believed that that is carried out primarily by mitogen-activated protein kinase (MAPK), which phosphorylates cPLA2 at the Ser505 residue in the flexible linker (Figure 12) [37]. In vitro studies showed that phosphorylation of cPLA2 enhances its activity by increasing the membrane residence time and the binding affinity of the cPLA2 to the perinuclear membrane, probably through a change of the catalytic domain conformation [35]. MAPK- and Erk1/2-dependent cPLA2 activation was shown to occur in smooth muscle, when stimulated by muscarinic receptors m1 and m2 [38]. In macrophages and neutrophils, colony-stimulating factor (CSF1) stimulates MAPK to phosphorylate cPLA2 [39]. In case of patients with Alzheimer’s disease, it was shown that accumulation of oligomeric amyloid-beta induces the MAPK-mediated activation of brain cPLA2 in a spatial-specific manner [40]. Protein kinase C (PKC) is another important kinase that regulates cPLA2 (Figure 12B) [41]. PKC can stimulate cPLA2 phosphorylation through both ERK-dependent and ERK-independent pathways. However, it was shown that the ERK-dependent pathway alone is not sufficient to activate cPLA2 and elicit AA release [41]. Further studies in canine kidney cells (MDCK-D1) showed that PKC plays a dual role in cPLA2 phosphorylation. The PKCα (one of the isoforms of PKC) can phosphorylate cPLA2 directly and release AA independent of MAPK, while the other isoforms of PKC (β1, β2, ɣ) can phosphorylate the MAP kinase, which will subsequently phosphorylate cPLA2. The P_2U_ receptor, which is a G-protein couple receptor (GPCR) in the MDCK-D1 cells, activates PKC, which subsequently activates MAPK to phosphorylate cPLA2 [42].

Ceramide-1-phosphate (C1P) is a bioactive sphingolipid synthesized by ceramide kinase by phosphorylating ceramide [43]. C1P plays an important role in cPLA2 activation in a Ca^2+^- and lipid-specific manner, being a mediator of arachidonic acid release. C1P triggers the translocation of the C2 domain of the cPLA2 enzyme into the perinuclear membrane from the cytosol [44,45]. During inflammatory responses to cytokines such as interleukin-1β, sphingomyelin is hydrolyzed by sphingomyelinase (a specific form of PLC) to form ceramide. Sphingolipids are phospholipids that reside mostly on the outer leaflet of the bilayer membrane. These phospholipids contain highly saturated acyl chains, which pack tightly in the lipid bilayer. In addition, cholesterol mixes with the sphingolipids in the bilayer membrane and increases the firmness of the outer surface, decreasing membrane permeability. On the other hand, ceramide mixes poorly with cholesterol and has a tendency to self-associate and segregate into microdomains. Thus, ceramide reduces the local rigidity of the membrane and increases the penetration ability of the cPLA2 into the local membrane environment via association of C2 domain with the C1P [46]. 

The phosphatidylinositol 4,5-bisphosphate (PIP2) hydrolysis pathway is an important pathway that can regulate the activation of cPLA2 both directly and indirectly. PIP2s are minor phospholipids distributed predominantly in the plasma membrane. These PIP2s are hydrolyzed by phospholipase C (PLC), forming diacyl glycerol (DAG) and inositol triphosphate (IP3), both acting as secondary messengers in the cell. IP3 diffuses into the cytoplasm and triggers the release of Ca^2+^ from ER, while DAG migrates laterally in the membrane and activates membrane-associated protein kinase C (PKC) in tandem with cytosolic Ca^2+^ ions. PKC activates MAPK, which phosphorylates cPLA2 already translocated to the membrane in response to elevated [Ca^2+^] [47,48]. Interestingly, PIP2s can also regulate cPLA2 directly after its translocation to the membrane by binding to the catalytic domain of the cPLA2 and increasing its catalytic activity dramatically. A cluster of cationic amino acid residues Lys-541, Lys-543, Lys-544 and Arg-488 in the catalytic domain of cPLA2 consists of the key residues that are believed to interact with anionic PIP2. Mutating these amino acids was shown to inhibit specific activation of cPLA2 via PIP2 binding [49]. The mutation obviously does not impair the calcium-induced translocation of the cPLA2 to the membrane. A study showed that the presence of PIP2 1 mol% can increase the binding affinity of cPLA2 for its substrate up to 20-fold. The study also demonstrated that in an in vitro setting, the PIP2 can also increase cPLA2 activity in a Ca^2+^-independent manner, while decreasing the requirement of Ca^2+^ for activation of cPLA2 [50,51]. 

## 4. Ca^2+^ Independent PLA2 (iPLA2)

Group VI phospholipase A2 enzymes are members of the PLA2 superfamily that are characterized as Ca^2+^-independent PLA2 (iPLA2) enzymes. The iPLA2 enzymes have a preference to hydrolyze phospholipids at the sn-2 position; however, they also show PLA1, lysophospholipase, transacylase and PAF acetylhydrolase activity as well [13]. There are six members in group VI PLA2 (A, B, C, D, E, F), which are also denoted as iPLA2β, iPLA2γ, iPLA2 δ, iPLA2ε, iPLA2ζ and iPLA2η, respectively. Their molecular weight ranges from 27 kDa (iPLA2ƞ) to 146 kDa (iPLA2δ) [6]. iPLA2β was the first member of this family of enzymes, which was isolated, purified and characterized from the cytosol of murine’s macrophages in 1994 [52]. iPLA2β is ubiquitously found throughout the mammalian body and it is highly expressed in testis and the brain. The cellular localization of iPLA2 varies based on the isoforms. For instance, iPLA2β is found intracellularly in the pancreas islets and B lymphocytes, while iPLA2ɣ is membrane-associated and found in the heart and skeletal muscles [13]. iPLA2δ is localized in the ER and Golgi complex of the neurons, iPLA2ε and iPLA2ζ are localized in the liver’s adipocytes, white and brown fat adipocytes, respectively, whereas iPLA2ƞ is ubiquitously spread in all body cells [53]. These enzymes are known to be regulated by ATP binding, calmodulin (CaM) binding and by caspase cleavage of the ankyrin repeats [53]. The iPLA2α is patatin and patatin-like homolog found in the potato tube [13]. The iPLA2β (group VI A) gene found in the human genome has multiple splice variants such as A-1, A-2, A-3, Ankyrin-1 and Ankyrin-2. Among them, at least two isoforms, namely A-1 and A-2, are active in the human body [6]. 

### 4.1. iPLA2 Crystal Structure

**Figure 13:**
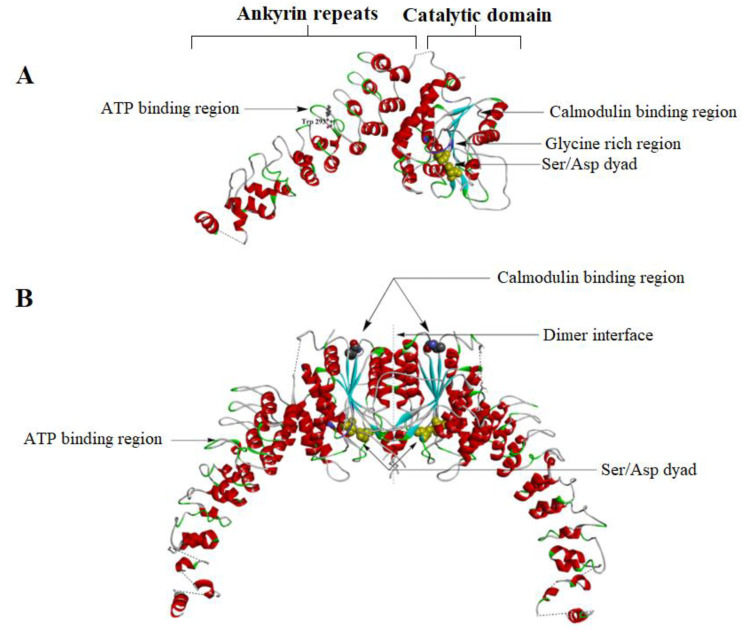
(**A**) The crystal structure of the monomeric form of iPLA2β (PDB- 6AUN). The ankyrin repeats (ANK) are shown on the left side and the catalytic domain on the right side. The linker between the ANK and the CAT (connected by a dashed line) is unresolved due to less electron density. Near the 6th ankyrin repeats, there is the Trp293 residue which is believed to be the ATP binding site. The catalytic dyad (Ser465 and Asp598) is shown in purple in the catalytic domain. The glycine-rich region, which forms a rigid handle, is shown in blue. Earlier studies showed it as the ATP binding domain. The CaM binding region is near residue 630. (**B**) The dimeric structure of the iPLA2. One CaM molecule binds two iPLA2 molecules and stabilizes the close confirmation of the active sites of the enzyme. Note that the two active sites are located near the dimeric interface (Image generated using BIOVIA Discovery studio).

The crystal structure of iPLA2β was solved recently by Korolev’s team (Figure 13A) [54]. Thus, the iPLA2 structure can be divided into three large segments: the N terminal domain, nine ankyrin repeats and a catalytic domain (CAT) (Figure 13A). The ankyrin repeat (ANK) is a motif structure containing 33 amino acid residues making a helix-turn-helix structure followed by a hairpin loop [54]. Interestingly, only the two variants of iPLA2β (VI-A1, VI-A2) have these ankyrin repeats, while the other isoforms do not have any ankyrin repeats [53]. The ankyrin repeats (ANK) interact with the integral membrane proteins such as ion channels and cell adhesion/signaling molecules. This explains the different cellular localization of iPLA2β in a tissue-specific manner. Unlike cPLA2, the iPLA2 enzymes do not have any CaLB domains. The translocation of the iPLA2 from the cytosol to the membrane is believed to be performed through the interaction of the ankyrin repeats with the membrane-bound proteins [13]. The ANK also has an ATP binding domain, which helps in regulating the activity of the iPLA2. The ANK domain is attached to the CAT domain at the opposite side of the membrane-binding surface. The linker between the ANK and CAT is unresolved due to poor electron density. The seventh and eighth ankyrin repeats form hydrophobic interaction with the CAT domain [54]. The catalytic domain forms a tight dimer between two iPLA2 molecules (Figure 13B). The catalytic domain has a large hydrophobic interface (~2800 Å), which triggers the dimerization of protein. The catalytic domain has an active site containing a Ser465/Asp598 dyad. The active sites of the dimeric form of the enzyme are closely located to the dimer interface and in proximity to each other (Figure 13B) [54]. 

### 4.2. iPLA2 Regulatory Mechanisms

One of the mechanisms by which iPLA2s are regulated is via ATP binding, which stabilizes their structure and activates them. The iPLA2β isoform is the only one that has been reported to be regulated by ATP binding [6]. An older study proposed that the ATP binding site is in the catalytic domain of the iPLA2, which has a highly conserved glycine-rich motif (GXGXXG) (Figure 13A) [13]. However, a recent crystallography study of the structure of the iPLA2 revealed that the ATP binds to the sixth ankyrin repeat (AR6) near the Trp293 residue and has a different conformation than the other ankyrin repeats [54,55]. iPLA2β expressed in Chinese Hamster Ovary (CHO) cells increases its activity 2–4-fold upon binding with ATP [13]. 

Another way of modulating the activity of iPLA2s is through caspase cleavage of the ankyrin repeats. Caspase-3 is a protease enzyme of the caspase superfamily. During cell apoptosis, caspase-3 is activated by caspase-8, -9 and -10. Activated caspase-3 truncates the ankyrin repeats of the iPLA2 and yields ~70 kDa iPLA2. This truncated iPLA2 shows increased catalytic function compared to the untruncated version. Increased activity of iPLA2 leads to a higher generation of lyso-PL, which acts as a signaling molecule on the surface of the apoptotic cells to be recognized and phagocytosed by macrophages. Interestingly, the caspase-3 can also cleave cPLA2, but in that case, it leads to the inactivation of the cPLA2 [56]. 

Calmodulin (CaM) binding is another way of regulating iPLA2. CaM inhibits the activity of iPLA2 in the presence of Ca^2+^. CaM is also essential for dimerization of iPLA2β monomers (Figure 13B) [57]. A single CaM can bind with the two molecules of the iPLA2 dimer and changes the conformation of the dimerization interface, together with both active sites (Figure 13B). CaM stabilizes the closed conformation of the active site, which does not allow any lipid substrate to enter the active site. The calmodulin binding domain of iPLA2β contains multiple contact points in the C-terminal region near residue 630 of the enzyme. CaM forms a catalytically inactive ternary complex called CaM/Ca^2+^/iPLA2 in the presence of calcium and protects against proteolysis. Dissociation of the CaM from the iPLA2 opens up the active site and allows lipid substrates to enter the active site [54]. 

### 4.3. Mechanism for Catalysis for cPLA2 and iPLA2

**Figure 14:**
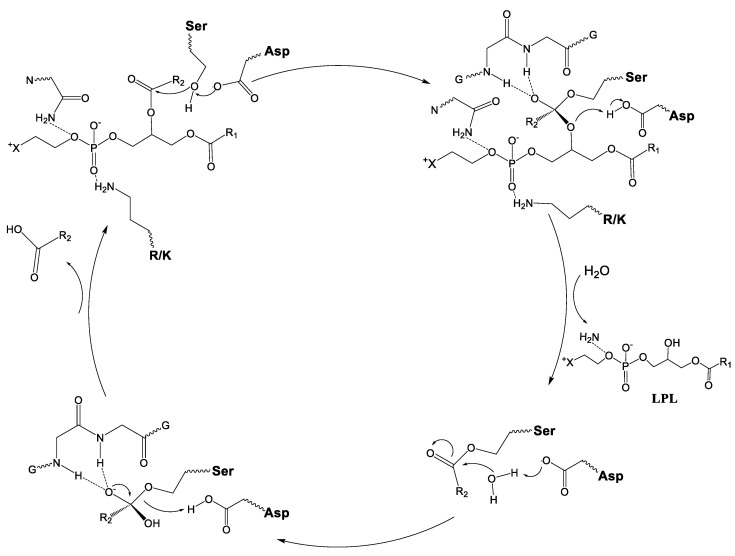
The proposed common mechanism of cPLA2 and iPLA2 for hydrolysis of phospholipids. The two classes of PLA2s share a common Ser/Asp dyad.

**Figure 15:**
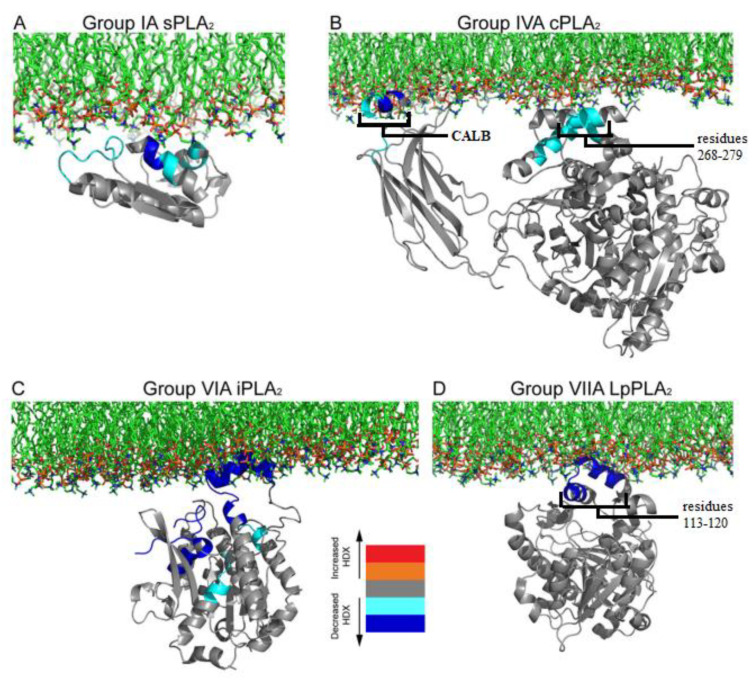
Proposed schematic models of interfacial binding of four different families of PLA2s. HDX-MS technique was used to study the interaction of monomers of different PLA2s bound to membranes, using crystal structures of (**A**) group IA sPLA2 (PDB- 1PSH), (**B**) group IV cPLA2 (PDB- 1CJY), (**C**) group VIA iPLA2 (homology model based on lipase patatin structure, PDB- 1OXW) and (**D**) group VIIA Lp-PLA2 (PBD- 3D5E). Reprinted from [59].

In contrast with sPLA2, cPLA2 and iPLA2 contain a Ser/Asp catalytic dyad instead of a His/Asp one. Therefore, both cPLA2 and iPLA2 share a common catalytic mechanism for hydrolysis (Figure 14) [58]. Once the phospholipid is inserted into the active site pocket of the enzyme, the phosphate group of the phospholipid is stabilized by an arginine (cPLA2) or by a lysine (iPLA2). The hydrolysis mechanism involves the deprotonation of the Ser by the Asp, followed by a nucleophilic attack of the OH^−^ to the ester bond to generate a tetrahedral intermediate B (Figure 15). The tetrahedral intermediate is stabilized by the oxyanion hole, formed by two glycines. The tetrahedral intermediate eliminates the lyso-PC, protonating it from the Asp. The Asp again removes a proton from a water molecule forming a hydroxyl group, which attacks the ester bond between the Ser and fatty acid (FA), generating a second tetrahedral group (D). The second tetrahedral group eliminates the FA, which leaves the active site and the Ser takes an H^+^ from the Asp, regenerating the initial active form of the enzyme [58]. 

## 5. Comparison of Membrane Surface Interaction within Four Major Classes of PLA2

The hydrogen/deuterium exchange mass spectrometry (HDX-MS) technique can be used to study the dynamic of protein/membrane interaction. The technique monitors the exchange rate of hydrogen with deuterium atoms in the amino acids of the protein backbone. The H/D exchange rate decreases dramatically when there is any interaction between the amide hydrogen with the lipid substrate. This technique was applied to reveal how four PLA2 families interact with the membrane (Figure 15) [59]. 

Panel A of Figure 15 presents the result of the HDX-MS study of the dynamic interaction of sPLA2-IA with the membrane bilayer, using DMPC lipid vesicles [60]. Whenever the hydrogen/deuterium (H/D) exchange rate of any amino acid decreases, it is depicted in blue. One can observe that the H/D exchange rate of the residues Tyr-3, Trp-61, Tyr-63, Phe-64 and Lys-6 decreases when the sPLA2 comes in contact with the DMPC bilayer. These aromatic residues are believed to be inserted into the lipid phase and to facilitate the hydrophobic interaction between the protein and lipid acyl tails (see sPLA2 section above). Lys-6 has an electrostatic interaction with the polar headgroup of the lipid membrane surface. The panel B shows the HDX-MS study results for the interaction of cPLA2 with the bilayer membrane surface [32]. cPLA2 has two distinct sites, a Ca^2+^ lipid-binding domain (CALB) and a catalytic domain. The HDX-MS technique revealed that both the CALB domain and catalytic domain interact with the phospholipid surface. The CALB domain (residues 35–39 and 96–98) contains hydrophobic residues, which can easily penetrate into the lipid phase. On the contrary, the catalytic domain (residues 268–279 and 466–470), which makes a ‘cap’ structure (discussed in Figure 11), binds only superficially through electrostatic interactions with phospholipids of the membrane [32]. 

The results of the HDX-MS study for the interaction of iPLA2 with the phospholipid substrate are shown in Figure 15C. The iPLA2 structure with the catalytic domain shown in panel C is not an actual depiction but rather a homology model based on the homolog structure lipase patatin (40% sequence similar to iPLA2). The interaction was studied between iPLA2 and 1-palmitoyl 2-arachidonoyl-phosphatidylcholine (PAPC) lipid vesicles. The H/D exchange rate levels indicate that only the catalytic domain interacts with the lipid membrane. Ankyrin repeats (not shown, for clarity) were not involved in the protein/lipid interaction. The hydrophobic region (shown as deep blue residues in Figure 15C) in the catalytic domain (residue 708–730) would penetrate the membrane surface, and the residues 631–655, 658–664 and 773–778 that had electrostatic interactions with the charged headgroups of the phospholipids to facilitate the protein/membrane interaction are also shown in light and deep blue [55,59]. 

GVIIA PLA2, also known as lipoprotein-associated PLA2 (Lp-PLA2) because of its association with LDL and HDL in human plasma, hydrolyzes the oxidized phospholipids. These Lp-PLA2 have substrate specificity for the acetyl group of platelet-activating factors (PAF). Lp-PLA2 binding with DMPC lipid vesicles was also analyzed by the HDX-MS method (Figure 15D). These studies showed that Lp-PLA2 residues 113–120 (shown as deep blue residues in the figure), which form an α-helix, have a decreased level of H/D exchange rate in the presence of DMPC lipid vesicles. These residues seem to play an important role in Lp-PLA2/LDL association. This α-helix contains hydrophobic residues such as Trp-115 and Leu-116, which contribute in the hydrophobic interactions of the enzyme with the lipid membrane [59,61]. 

## 6. Physiologic and Pathologic Role of PLA2s

The PLA2 superfamily of enzymes is ubiquitously expressed throughout the body and plays a wide variety of roles both in regular physiology and pathologic conditions of the human body. A brief description of certain roles of PLA2 in the human body are discussed below. 

### 6.1. sPLA2 Role in Physiology and Pathology

sPLA2s are secreted outside the cells and require Ca^2+^ for their activity. They primarily target substrates located outside the cell [62]. They also tend to show similar substrate specificity based on the groups. For instance, the group II subfamily of sPLA2 can preferentially hydrolyze phosphatidylethanolamine better than phosphatidylcholine, whereas groups III, V and X have a higher tendency of preferentially hydrolyzing phosphatidylcholine. While sPLA2- IB, IIA and IIE do not show discrimination for sn-2 fatty acid, sPLA2-V prefers to hydrolyze lipids with unsaturated fatty acids in the sn-2 position having a low degree of unsaturation, such as oleic acid (C18:1) and linoleic acid (C18:2). sPLA2-IID, IIF and X have a preference for processing lipids with PUFAs in the sn-2 position, such as ω6 arachidonic acid (C20:4) and ω3 docosahexaenoic acid (22:6) [62]. 

**Figure 16:**
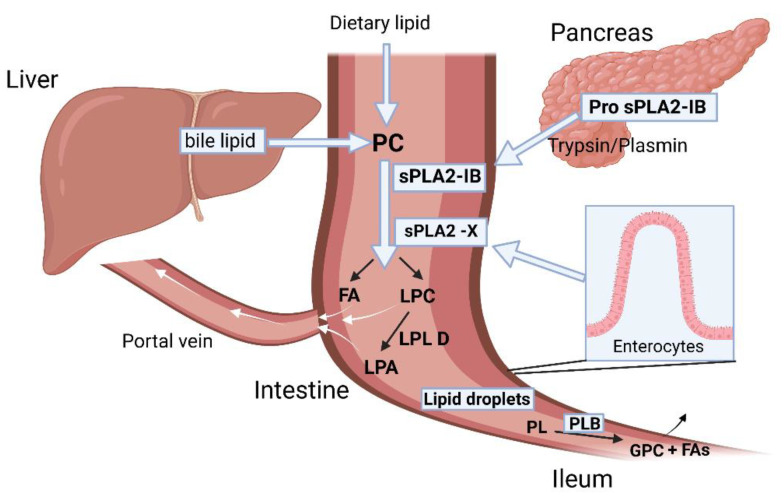
Physiologic role of phospholipases in GI tract. Pancreatic sPLA2-IB is secreted in pancreatic juice as a zymogen and activated by trypsin or plasmin in the intestinal lumen. sPLA2-X is secreted by the enterocytes of the intestinal lumen. Both pancreatic sPLA2-IB and enterocytic sPLA2-X hydrolyze dietary and bile-secreted phospholipids. Phospholipase B is secreted in the distal intestine and hydrolyzes the undigested phospholipids yielding glycerylphosphocholine and FAs. Created with BioRender.com.

sPLA-IB, the pancreatic PLA2, is synthesized in the pancreatic acinar cells and is secreted as inactive zymogen into pancreatic juice (Figure 16). It becomes activated in the presence of trypsin/plasmin and digests both dietary phospholipids and bile-secreted phosphatidylcholine (PC) in the intestinal lumen, producing lysophosphatidylcholine (LPC) and free FA [63]. The free fatty acids are absorbed through the intestinal membrane. The LPC can be further hydrolyzed into lysophosphatidic acid and choline by lysophospholipase D. In addition, the LPC can also be passively absorbed into the enterocytes, where they can play a role in intracellular lipid trafficking required for chylomicron assembly and secretion [64,65]. Two other important phospholipases expressed in the GI tract are sPLA2-X and PLB (phospholipase B). sPLA2-X is expressed in the enterocytes of the proximal intestine and works along with the pancreatic sPLA2-IB to hydrolyze PC to produce LPC and FAs. Phospholipase B (PLB), on the other hand, is expressed in the distal intestine (Ileum) and hydrolyzes the remaining phospholipids at both sn-1 and sn-2 fatty acyl chain positions and generates glycerophosphorylcholine (GPC) and FAs [66].

**Figure 17:**
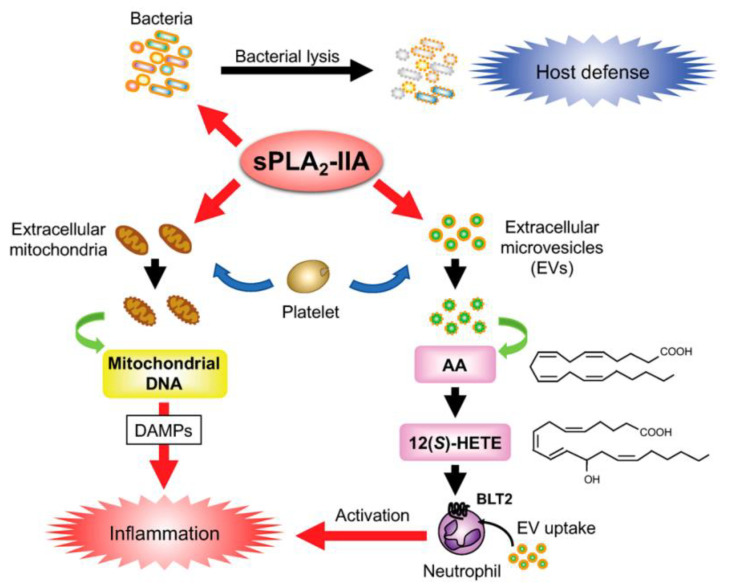
Physiological roles of sPLA2-IIA, as a key player in host defense against foreign self-assembled lipid structures in the blood, through the degradation of bacterial membranes, and in inflammation, where it hydrolyzes arachidonic acid from extracellular micro-vesicles, which subsequently feeds the inflammatory pathway. It also cleaves cardiolipin from extracellular mitochondria (released from activated neutrophils, platelets, mast cells, lymphocytes, etc.), liberating mitochondrial DNA that potentiates inflammation [62].

sPLA-IIA is often referred to as ‘inflammatory sPLA2’ because of its increased expression induced by proinflammatory responses in a variety of cells and tissues such as macrophages, monocytes, T-cells, mast cells, neutrophils and also in the synovial fluids [6,62,63]. Activated platelets or leukocytes at the inflammatory sites can release vesicles such as exosomes or micro-vesicles, which are commonly known as extracellular vesicles (EV) (Figure 17). These EVs are found in blood plasma, tears, synovial fluid and in bronchoalveolar lavage fluid [67]. The EVs contain a large variety of phospholipid substrates, which are hydrolyzed by the sPLA2s. Thus, it was shown that sPLA2-IIA can hydrolyze the phospholipids of the EVs present in the synovial fluid of rheumatoid arthritis patients and can release arachidonic acids (AA). AA is subsequently processed by COX enzyme, (Figure 1), generating inflammatory eicosanoinds that further enhance local inflammation. AA can also be metabolized by platelet-type 12-lipoxygenase to 12-hydoxyecosatetraenoic acid (HETE) leading to another inflammatory pathway [62].

The extracellular mitochondrial membrane is another target of hydrolysis by sPLA2 enzymes. Group IIA sPLA2 are highly potent in hydrolyzing the mitochondrial membranes releasing AA, lyso-cardiolipin and also encapsulated mitochondrial DNA, which can promote inflammation. The extracellular mitochondria can be released from activated cells such as neutrophils, platelets, mast cells, lymphocytes, hepatocytes and sometimes from damaged tissues or organs as well. Mitochondrial damage-associated molecular patterns (DAMP) (Figure 17), a proinflammatory mitochondrial component, were identified in pathological conditions such as rheumatoid arthritis, burn injury and systemic lupus erythematosus [67]. 

sPLA2-IIA also plays a major role in host defense via its antimicrobial activity. It degrades bacterial membrane by hydrolyzing phosphatidylethanolamine and phosphatidylglycerol, which are abundant in the bacterial membrane (Figure 17) [62]. GIIA-sPLA2 is highly expressed in human tears, where it plays a bactericidal role [68]. Blood plasma concentration of sPLA2-IIA can increase up to 500-fold in patients with acute diseases such as sepsis, peritonitis and bacterial infection, compared to healthy persons [69].

The mechanisms of AA release by sPLA2 have been discussed in detail by earlier reviews [6,26]. sPLA2 primarily releases AA by heparan sulfate proteoglycans (HSPG) dependent and independent pathways. HSPG are glycoproteins found on the cell surface, which can initiate multiple cell functions, such as cell adhesion, signaling and endocytosis [26]. In case of HSPG-dependent mechanism, sPLA2-IIA that binds with the cell surface anionic HSPG becomes internalized into an intracellular vesicular compartment of the activated cells through a caveolae-dependent endocytic pathway [70]. A study shows that glycosylphosphatidylinositol (GPI)-anchored HSPG acts as an adaptor for sPLA2-IIA, which facilitates the trafficking of sPLA2-IIA into the subcellular compartments of the cells and releases AA from those compartments [70]. On the contrary, sPLA2 isoforms such as IB, IIC and IIE that weakly bind with the heparinoids have failed to release AA from the cellular compartments. Furthermore, sPLA2-X, which does not bind to heparin, can liberate AA from the cell’s plasma membrane rich with PC on the outer leaflet [71]. 

Secreted PLA2s, especially groups IIA, V and X, can play an important role in atherosclerosis. A study showed that young adults (age 24 to 39 years) with cardiovascular disease had an increased level of GIIA sPLA2 in their blood plasma, which may contribute to atherogenicity [72]. Transgenic mice, which expressed group IIA sPLA2, had an increased atherosclerotic lesion compared to the non-transgenic littermate’s mice, irrespective of their feeding habit. In the same study, sPLA2-IIA transgenic mice exhibited a lower level of high-density lipoprotein (HDL) and a higher level of low-density lipoprotein (LDL). One of the possible mechanisms for atherosclerosis would be the interaction of sPLA2 with those lipoproteins [73]. 

### 6.2. Role of sPLA2 in Cancer

sPLA2s are widely known to be overexpressed in various tumors and cancer cells [3]. They play pro-tumorigenic roles in colon [74], breast [75,76], lung [77], ovarian [78,79] and prostate cancer [80]. However, they have an antitumor effect in gastric cancer [81]. Among sPLA2, groups IIA and X show high expression and enzymatic activity related to cancer progression. Group IIA sPLA2 can hydrolyze cellular phospholipids and release arachidonic acid (AA) and lysophosphatidic acid (LPA). AA can be further metabolized into prostaglandins and leukotrienes and can contribute to cellular proliferation and tumor progression, as outlined above. LPA can stimulate cell proliferation, tumor invasion and differentiation [82]. 

Thus, sPLA2-IIA was found to be overexpressed in human breast cancer tissues and was identified in nipple aspirate fluid (NAF) [83] and in blood serum from breast cancer patients [84]. Group IIA sPLA2 concentration in NAF samples from breast cancer patients was positively correlated with the tumor stage, with higher expression seen in stages III and IV, as compared to stage I and II cancers [83]. sPLA2 as a whole exhibits differential expression in various cell lines of breast cancer, suggesting that different groups of sPLA2s may play different roles based on breast cancer subtypes [85]. 

In a study involving healthy individuals in the age group 18 to 63, it was shown that they had an average serum level of group II PLA2 of 2.2 ± 0.1 ng/mL, with a range of concentration of 1.4–4.2 ng/mL. However, group II PLA2 levels increased significantly in the serum of various cancer patients in a similar age group. The average serum level of group II PLA2 in patients with cancers: lung (9.5 ± 4.0 ng/mL), breast (9.1 ± 3.8 ng/mL), esophagus (8.0 ± 2.8 ng/mL), colon (13.1 ± 4.4 ng/mL) liver (31.4 ± 12.5), pancreas (9.7 ± 3.2 ng/mL) and bile duct (9.2 ± 3.3 ng/mL) [84]. 

### 6.3. Physiologic and Pathologic Role of cPLA2

Cytosolic PLA2s are ubiquitously expressed throughout the cells of the whole body. They play a major role in inflammation by hydrolyzing phospholipids and releasing arachidonic acid and lysophospholipids. cPLA2α shows high substrate specificity for phospholipids containing AA. Upon hydrolyzing phospholipids at the sn-2 position, cPLA2 releases arachidonic acid inside the cell, which can be further processed by either cyclooxygenase (COX) or lipoxygenase (LOX) pathways and generate prostaglandins, thromboxanes and leukotrienes, as shown before (Figure 1). Lysophospholipids are also produced through cPLA2-mediated phospholipid hydrolysis and can act as second messengers for GPCR signaling [6]. 

cPLA2 are localized on the surface of the endoplasmic reticulum and Golgi complex in the epithelial cells. They regulate the formation of tubules, Golgi structures and intra-Golgi transport. Upon binding on the Golgi structure, cPLA2 releases FA and lysophospholipids. Lysophospholipids are believed to affect the positive curvature of the Golgi membrane, which lead to the formation of tubules, which cause Golgi stacking and promote intra-Golgi transport. cPLA2 also regulates the transport of tight junction and adherent junction proteins from the Golgi complex in confluent endothelial cells to cell contacts. Upon cellular activation, the intracellular concentration of Ca^2+^ increases and cPLA2α translocates to the perinuclear membrane of Golgi and endoplasmic reticulum. The enzyme is activated by phosphorylation through mitogen-active protein kinases (MAP kinases). Phosphatidylinositol bisphosphate (PIP2) and Ceramide-1-phosphate (C1P) help in subcellular localization and activation of cPLA2α to stimulate the production of eicosanoids in vitro [62]. 

cPLA2s are expressed in liver cells and adipose tissue and regulate lipid storage in those cells. cPLA2s are also expressed in brain cells, where they play an important role in neuronal homeostasis. They can play a critical role in diseases such as spinal cord injury, neurodegenerative diseases and postischemic brain injury [86]. 

Alzheimer’s disease (AD) is a neurological disorder, commonly characterized by an increased accumulation of β-amyloid (Aβ) plaques and neurofibrillary tangles in the brain [87]. Aβ is produced from the amyloid precursor protein. The soluble amyloid-beta peptides contribute in the early development of the AD such as synapse loss and cognitive impairment in patients [88]. The oligomeric form of Aβ is the most aggressive form, which interferes with the synaptic transmission in the neurons and induces inflammatory pathways. Aβ peptides open up calcium ion channels, leading to an increased concentration of intracellular Ca^2+^ ions, which trigger inflammation, cellular dysfunction and cell death. Aβ peptides also induce MAPK pathways, which activate cPLA2 via phosphorylation and translocate them from cytosol to the membrane system [40,89]. Increased activity of the cPLA2 enzyme contributes to the progression of the AD pathway mainly through the release of arachidonic acid. AA is transformed by COX and LOX enzymes into lipid mediators such as leukotrienes, prostaglandins, lipoxins and hydroperoxy acids, which lead to inflammation [90]. NFκB, a transcriptional factor present in neurons, astrocytes and microglia, is activated by Aβ through an oxidative pathway involving reactive oxygen intermediates in AD patients [91]. NFκB upregulates cPLA2 expression in microglia, resulting in increased NADPH oxidase activity, production of superoxide, PGE_2_, and nitric oxide (NO) in microglia. cPLA2 also reduces microglia’s ability to phagocyte Aβ in the AD brain, which further promotes disease pathogenesis [92]. In addition, genetically silencing cPLA2 in a mouse model of Alzheimer’s disease was shown to help in reducing Aβ-induced neurotoxicity and improve the cognitive function of the animals [93].

Cdk5, a cyclin-dependent kinase localized in the brain, is a key regulator of the architecture of this organ. Cdk5 is involved in brain development, including higher cognitive functions such as learning and memory functions. It was found that Cdk5 is deregulated in neurodegenerative diseases such as Alzheimer’s, Parkinson’s and Huntington’s [94]. Another study has shown that Cdk5/p25 (p25 being an activator of Cdk5 protein) increases neurotoxicity in the Alzheimer’s patient’s brain. Cdk5/p25 upregulates cPLA2 expression in microglial cells to release lysophosphatidylcholine (LPC), which causes astrogliosis, increased production of cytokines and subsequent generation of neuroinflammation and neurodegeneration [95]. 

### 6.4. Role of cPLA2 in Cancer

cPLA2 plays an important role in inflammation and cancer-related diseases through the release of arachidonic acids, which contributes to carcinogenesis. In most cancers, cPLA2 plays a pro-tumorigenic role, except for colon cancer, where downregulating cPLA2 was shown to significantly suppress the formation of tumors in the intestine [3]. 

cPLA2 is regulated by the mitogen-activated protein kinase (MAPK) pathway, which is a tyrosine kinase receptor. Transforming growth factor-β (TGF-β) initiates cPLA2 phosphorylation through the MAPK pathway (via p38 and ERK), which leads to increased production of AA-derived eicosanoids in the cancer cells [96]. Subsequently, the eicosanoids activate the GPCR-mediated signaling pathways and promote transcription, translation and release of cytokines such as TNF-α and IL6s. These cytokines induce NF-κB-dependent gene expression, which further upregulates the expression of cPLA2s and sPLA2s. Thus, the AA release cycle continues, and tumor cells proliferate [97,98].

Cytosolic phospholipase A2α (cPLA2α) is commonly known to be overexpressed in human breast cancer (BC) tissues, where it plays a role in cancer metastasis. Studies conducted in the breast cancer cell lines MDA-MB-231 and T47D have shown that cPLA2 significantly increased the migration and invasion of breast cancer cells. Suppressing the cPLA2 gene reduced tumorigenesis and cancer metastasis [99]. cPLA2 has also been proposed as a potential biomarker in different breast cancer subtypes. A fluorescent-tagged arachidonic acid substrate was used to measure the enzyme activity of the cPLA2 through fluorescent microscopy. Different breast cancer cells express cPLA2 at different levels, with the highest level in the basal-like triple-negative cells, followed by the HER2 cells and the lowest in the luminal-like cells [100]. cPLA2 expression is strongly correlated with the mTOR (mammalian target of rapamycin) signaling pathway. The mTOR signaling pathway is upregulated in many cancers and plays a crucial role in the development of tumors, including breast cancers. However, the tumorigenesis mechanism is not clear. It was proposed that the increased release of arachidonic acid activates mTOR, which subsequently leads to angiogenesis and BC tumorigenesis [101].

### 6.5. Physiologic and Pathologic Role of iPLA2s

iPLA2s play a crucial role in homeostasis and phospholipid remodeling of the membrane during cell-cycle progression. These enzymes are also involved in cell proliferation, apoptosis, bone formation, sperm development, cardiolipin acetylation, glucose-induced insulin secretion and monocyte recruitment. In addition to phospholipase A2 activity, iPLA2s also exhibit lysophospholipase and transacylase activity [13,102,103]. iPLA2β and iPLAγ are membrane-associated, localized on the mitochondria and peroxisome and help maintain the integrity of these organelles, preventing them from rupturing. iPLA2β can regulate diabetes through its contribution to beta-cell apoptosis, superoxide production and glucose-induced RhoA/Rho-kinase activation. iPLA2δ (group VI C) is the largest protein among the iPLA2s. It is a transmembrane protein that is expressed in neurons. The iPLA2δ enzyme is localized on the endoplasmic reticulum and Golgi apparatus of the neurons and exhibits esterase activity and important regulatory properties. Groups VI D, VI E and VI F are smaller iPLA2s localized in the adipose tissues. They exhibit triacylglycerol hydrolase and transacylase activity in adipocytes [6].

#### Role of iPLA2 in Myocardial Ischemia

Myocardial ischemia (MI) occurs when the blood flow to the heart muscle is obstructed due to partial or complete blockage of the coronary arteries. iPLA2β (also known as group VIA PLA2) regulates pathophysiology during myocardial ischemic conditions. However, the mechanism for iPLA2β contribution in MI is poorly understood [104]. Multiple factors are involved in MI, such as oxidative stress, increased intracellular Ca^2+^ concentration and inflammation, which disturb ER normal function and cause ER stress, a condition which hampers ER protein folding ability and accumulates misfolded protein in the ER [104]. A recent study has shown that iPLA2β is overexpressed during myocardial ischemia, which induces ER stress by translocating iPLA2β to the ER and causing apoptosis. Inhibiting iPLA2β improves ER stress and decreases cell death [104]. iPLA2γ is another group of iPLA2s, which are membrane-associated enzymes localized on the mitochondria and peroxisomes. iPLA2γ KO mice have shown decreased production of oxidized fatty acid in the ischemic cells and reduced the infarct size in this animal model of MI [105]. Furthermore, a study in the rabbit myocardium has shown that catalytically active iPLA2 was localized on the outer face of the inner mitochondrial membrane. It was hypothesized that during MI, increased activity of iPLA2 led to accelerated catabolism of mitochondrial phospholipids, which caused the membrane to lose Ca^2+^ homeostasis, permeability transition and release of cytochrome c in the mitochondria, which further potentiated the apoptotic pathway. Inhibition of the iPLA2 using an iPLA2-specific inhibitor, bromoenol lactone (BEL), was proven to be cardioprotective and to significantly decrease the infarct size [106]. 

### 6.6. Role of iPLA2 in Cancer

iPLA2s are overexpressed in ovarian cell lines such as SKOV-3 and Dov-13, and their overexpression is associated with enhanced cellular activity and cell growth [107]. One of the activation mechanisms for iPLA2 is caspase-3 via a proteolytic process, which results in an ankyrin-repeat domain truncated iPLA2. This shorter iPLA2 shows increased activity in generating arachidonic acid and lysophosphatidylcholine. Arachidonic acid enhances the migration of the cancer cells, and lysophosphatidylcholine activates Akt, resulting in the inhibition of apoptosis [108]. Another study showed that downregulating iPLA2β expression using RNA interference in a nude mice model of ovarian cancer halted the cancer cell proliferation and tumorigenicity [109].

To the best of our knowledge, no study case was conducted in breast cancer to assess the level of expression of iPLA2, unlike the sPLA2s. Several studies examined the influence of iPLA2 in breast cancer and attempted to correlate it with smoking. It was shown that cigarette smoking upregulates the expression of COX-2 and PGE2 synthase. These enzymes facilitate the metabolic conversion of arachidonic acid to prostaglandin E2. Smoking also increases the accumulation of PAF in breast cancer tissues. Together, PGE2 and PAF have a significant role in cancer progression. It was shown that inhibiting iPLA2 in the breast cancer tissues using an iPLA2-specific inhibitor rescinded the metastasis of the cancer cells [110]. Nicotine induces MMP-9 and iPLA2β expression in breast cancer, which facilitates cell proliferation and lung metastasis. Bromoenol lactone (BEL), a known iPLA2 inhibitor, was able to reduce nicotine-induced tumor growth [111]. Furthermore, iPLA2 deficiency in knockout mice inhibited the metastasis of breast cancer to the lung [112]. 

### 6.7. Physiologic and Pathologic role of Lipoprotein-Associated PLA2 (Lp-PLA2) 

Lp-PLA2s are unique members of the PLA2 superfamily. These Lp-PLA2s are assigned to groups VII and VIII. Both of them catalyze the hydrolysis of the acetyl group from the sn-2 position of PAF and produce lyso-PAF and acetate. Group VII-A hydrolyzes PC with shorter chain residues at sn-2 more efficiently than with PC with longer chains at the sn-2 position [6]. Lp-PLA2s are primarily secreted by the macrophages, which circulate in the blood-forming complex with LDL and HDL. These enzymes have a molecular weight of 45 kDa and have a characteristic GXSXG motif, which is also found in lipases and esterases. They have a catalytic triad of Ser/Asp/His. Lp-PLA2 lacks a lid region and has an open conformation of the active site, which can accommodate various sizes of sn-2 acyl chain in the phospholipid substrate [113,114]. Lp-PLA2- VII-A is secreted by the macrophage, monocytes, mast cells and T-lymphocytes, and its expression and secretion increase dramatically when the macrophages are activated by exogenous stimuli such as cytokines, TNFα, IFNɣ, etc. [14,113]. 

Lp-PLA2s can be used as a biomarker in cardiovascular diseases. They increase the risk of CVD and strokes by twofold [115]. Lp-PLA2s mediate vascular inflammation via regulating lipid metabolism in the blood, mainly in atherosclerosis. These enzymes hydrolyze the oxidized phospholipids on the LDL particles within the arterial intima and produce inflammatory mediators such as LPC and oxidized FA. Subsequently, the hydrolyzed products initiate a cascade of events leading to atherosclerotic plaque formation, such as upregulation of adhesion molecules and cytokines, differentiation of monocytes into macrophages and recruitment of macrophages into the arterial intimal space, which engulf the oxidized LDL [115,116]. Lp-PLA2s were found in the macrophages of both human and rabbit atherosclerotic lesions. High Lp-PLA2 activity was detected in the atherosclerotic aortas of hyperlipidemic rabbits compared to the normal aortas of control rabbits. Inhibiting Lp-PLA2 with a specific inhibitor, such as darapladib, significantly reduced Lp-PLA2 activity in the lesion and attenuated the progression of atherosclerotic plaque in the coronary arteries [116]. Group VII-B Lp-PLA2s are intracellular enzymes that are mainly expressed in the epithelial cells such as the ones from proximal and distal tubules of the kidney, intestinal tract and hepatocytes [117]. Lp-PLA2 VII-B overexpressed in CHO-K1 cells can suppress oxidative-stress-induced cell deaths. These enzymes are reported to function as anti-oxidant phospholipase and protect the cell against oxidative stress my hydrolyzing oxidized phospholipids [118]. They are also overexpressed in neurons when there is ischemic damage in the central nervous system. Group VIII Lp-PLA2s are intracellularly expressed in brain cells and are believed to play roles in brain development. They were also reported to be localized in the Golgi complex and to regulate the functional organization of the Golgi apparatus [6]. 

### 6.8. Physiologic and Pathologic Role of Lysosomal PLA2 (LPLA2)

LPLA2s are the members of group XV PLA2 known to be localized on intracellular vesicles such as lysosomes and endosomes [15]. Lysosomal PLA2 has a molecular weight of 45 kDa. These enzymes are ubiquitously expressed throughout the body but are predominantly found in the alveolar macrophages responsible for lung surfactant catabolism [119]. In some cases, LPLA2s have shown 50 times higher activity in alveolar macrophages as compared with peritoneal/peripheral macrophages. It is hypothesized that LPLA2s have both PLA2 and transacylase activity. It can transfer the acyl group from the sn-2 position of the phospholipid to the hydroxyl group of either the water molecule (phospholipaseA2 activity) or the ceramide molecule at C1 position (transacylase activity) [15]. LPLA2-deficient mice develop phospholipidosis in the lung—a lysosomal storage disorder characterized by increased accumulation of intracellular phospholipids [120]. Another important location of LPLA2 expression is in the ocular region. A study has revealed significantly higher activity of LPLA2 in a patient’s eye with glaucoma disease in comparison to other ocular diseases. LPLA2 metabolizes the undigested substances filtered on the trabecular meshwork (TM) and maintains the balance of intraocular pressure (IOP) between the aqueous humor and TM. However, the precise mechanism has not been clarified [121]. Another report revealed that endotoxin-induced uveitis in rats (an eye inflammation animal model) displayed increased activity of LPLA2 in the anterior chamber of the eye. While LPLA2 activity was correlated to the anterior chamber’s inflammation, LPLA2 activity was not significantly increased in the rat serum or in the cerebrospinal fluid. Samples collected from patients in the clinical settings with a history of uveitis showed significantly higher activity of LPLA2 than from senile patients with cataracts [122]. Cataract formation is a common complication in patients with uveitis, which results from chronic intraocular inflammation and the use of corticosteroids to treat the inflammation [123]. 

### 6.9. Physiologic and Pathologic Role of Adipose Specific PLA2 (Ad-PLA2)

Human AdPLA2s are expressed ubiquitously throughout the body but are predominantly found in the adipose tissue. They play an important role in obesity. AdPLA2 knock-out mice exhibit resistance to obesity induced by a high-fat diet. They further displayed high lipolytic activity in white adipose tissue as compared to the wild-type mice [8]. AdPLA2s have a catalytic triad of Cys–His–His, with a molecular weight of 18 kDa. They exhibit calcium-independent phospholipase activity for PCs and PEs and have a different physiological role than the sPLA2. Interestingly, AdPLA2 has been categorized as a class II tumor suppressor. A study showed that the AdPLA2 gene, also known as H-REV-107, is downregulated in ovarian carcinoma cell lines. It was also shown that expression of rat H-rev-107-1 gene resulted in growth inhibition of RAS transformed cells, both in vitro and in vivo [124]. A recent study has shown that AdPLA2 is expressed in the skeletal muscle and is downregulated in a peripheral artery disease condition. This may hamper the walking capacity due to its abnormal effects on skeletal muscle metabolism [125]. 

## 7. Conclusions

The superfamily of phospholipase A2 comprises a very diverse collection of members, evolved to perform the same hydrolytic reaction in different specific environments. We reviewed the diversity of its members, comparatively presenting their structure, mechanism of activation and of action and substrate specificity and relating these properties with subcellular localization and tissue distribution of the isozymes. We have also highlighted the role played by different PLA2s in key physiologic processes in the human body and in different pathologies, ranging from inflammation and cancer to Alzheimer’s disease, with the aim of providing an up-to-date overview of the impact of this class of enzymes in human health and diseases.

## Figures and Tables

**Table 1:** The main groups and subgroups of PLA2s.

Type	Group	Subgroup	Source	Molecular Weight (kDa)	Catalytic Residues	Substrate Specificity	Secreting Cells	Ref.
sPLA2	I	A	Cobras/kraits	13–15	His/Asp Ca^2+^ is required for substrate binding and catalysis	More active against zwitterionic PC		[6]
	I	B	Human/porcine pancreas	13–15			Pancreatic acinar cells	[9]
	II	A	Rattlesnake, human synovial fluid	13–15		High affinity for anionic phospholipids such as PE, PG, PS	Synovial fluid, tears, leukocytes, platelets, macrophages, monocytes, T-cells, neutrophils	[6,9]
	II	B	Gaboon viper	13–15				[7]
	II	C	Rat/murine testis	15				[7]
	II	D, E, F	Human/murine pancreas/brain/spleen/heart/uterus/embryo/testis	14–17			G-IID in lymph tissue DC; G-IIE in hypertrophic adipocytes. These are also expressed in macrophages and smooth muscle cells	[9]
	III		Lizard/bee	15–18		Sperm membrane phospholipid remodeling	CNS, peripheral, neural fibers, mast cells, epididymal epithelium	[9,10]
	V		Human/murine macrophage/heart/lung	14		High affinity for cell membrane PCs	Bronchial epithelial cells, macrophage, neutrophils, eosinophils, cardiomyocytes, hypertrophic adipocytes	[9,11]
	IX		Snail venom					[7]
	X		Human spleen/thymus/leukocyte	14		High activity toward PCs	Bronchoalveolar lavage fluid of asthmatic patients, airway epithelium, macrophages, smooth muscle cells, adrenal glands, pancreatic β cells, gut epithelium, sperm acrosome, hair follicles	[9]
	XI	A, B	Green rice shoots	13				[12]
	XII	A, B	Human/murine	19			Hepatocytes	[9]
	XII		Parvovirus	<10				[12]
	XIV		Symbiotic fungus/*Streptomyces*	13–19				[12]
cPLA2	IV	A, B, C, D, E, F	Human macrophage/pancreas/liver/brain/heart/skeletal muscle Murine placenta/heart/thyroid/stomach/testis/liver cells, neuronal cells	60–144	Ser/Asp Ca^2+^ is required for membrane binding and activation	High specificity for PC, PE containing arachidonic acid at sn-2 position	Intracellularly localized in endoplasmic reticulum, Golgi complexes in epithelial cells	[6,13]
iPLA2	VI	A, B, C, D, E, F	Human/murine	84–90	Ser/Asp Ca^2+^ is not required for catalysis	Phospholipase. Lysophospholipase activity on PL and lyso-PL, transacylase, acyl-CoA thioesterase activity	Group VI-A localizes on mitochondrial membrane and group VI-B on mitochondria and peroxisomes; group VI-C is expressed in neurons and localized on ER and Golgi body; groups VI-D, VI-E and VI-F are localized in adipose tissues	[6,13]
Lp-PLA2 (Lipoprotein- associated PLA2)	VII	A, B	Human, porcine, murine, bovine	40–45	Ser/His/Asp Ca^2+^ is not required for catalysis	Groups VII and VIII hydrolyze the acetyl group from sn-2 position of PAF and generate lyso-PAF and acetate. Group VII-A prefers PC with a shorter chain at sn-2 position	Group VII-A is secreted extracellularly by macrophages, monocyte, mast cells and T-cells. These are also associated with LDL and HDL in plasma; group VII-B is intracellularly expressed in epithelial cells of the kidney, intestine and hepatocytes. They are also overexpressed in neurons; group VIII is intracellularly expressed in brain cells	[6,14]
	VIII	A, B	Human	26–40				
LPLA2 (Lysosomal PLA2)	XV		Human, murine, bovine	45	Ser/His/Asp Ca^2+^ is not required for catalysis	LPLA2 is specific towards PC and PE. They hydrolyze the acyl chain at both sn-1 and sn-2 positions of PC and PE with a preference for sn-2 position	Highly expressed in alveolar macrophages; localized in lysosomes	[15]
Ad-PLA2 (Adipose tissue specific PLA2)	XVI		Human, mouse	18	Cys/His/His Ca^2+^ is not required for catalysis		Ubiquitously expressed throughout the body. Highly expressed in adipose tissue.	[6,16]

## Data Availability

The data presented in this review were compiled from the cited work. Details about the adapted figures and graphic are available upon request.
